# On the Effects Produced by Posture, on the Frequency and Character of the Pulse, in Health and in Disease

**Published:** 1831

**Authors:** 


On the Effects produced by Posture, on the Frequency and Character of the Pulse, in Health and in Disease. By Robert J. Graves, M. D.

[Dublin Hospital Reports, Vol. V.]

The influence of posture on the pulse has long been acknowledged; but hitherto not sufficiently investigated. Dr. Graves, therefore, has laudably dedicated a portion of his time and attention to the subject, and the paper now before us is the result.

"In healthy persons the pulse in the erect posture is more frequent than in the horizontal, by from six to fifteen beats in the minute. If the pulse is but sixty the difference is generally not more than six or eight, and this difference increases with the frequency of the pulse at the time of the experiment: thus if it has been raised to 90 or 100 by moderate exercise, it is not unusual to find the difference twenty or thirty.

As the muscular exertion necessary to keep the body in the erect posture, might be considered as the cause of this greater frequency, it became necessary to contrive means of placing the body in any desired posture, without the necessity of muscular exertion on the part of the subject of the experiment; this was effected, and it was found that when the posture was changed by means of such a contrivance the difference between the frequency in the horizontal and the erect postures, was not less than when muscular exertion was used.

I now anticipated, that if the body was placed with the head down and the feet up, a still further retardation of the pulse would be produced; it was, indeed, natural to conclude from the preceding experiments, that posture alone was the cause of the retardation observed in the body when placed horizontally, and consequently, that this effect would be augmented on still more depressing the head, and that the maximum of retardation would occur in the inverted position."

Here, however, experience did not confirm preconceived theory, for no retardation of the pulse was occasioned, nor yet acceleration. In the inverted position, the strength of the pulse was diminished, and often very considerably—a fact that may be explained by the weight of the blood pressing on the aortic valves, and thus necessarily opposing an unusual impediment to its egress from the left ventricle. The pulse is stronger in the horizontal than in the erect porture—consequently its maximum of strength and minimum of frequency are attained together. This, our author thinks, may account for the relief obtained by placing patients in the horizontal posture, in order to avoid syncope. In all other diseases in which Dr. G. has investigated this subject, he has found the difference between the frequency of the pulse in the erect, sitting, and horizontal postures; "*but in six cases of hypertrophy with dilatation of the heart, no such difference was perceptible, although all these patients, at the time of my making the experiment, were in a debi-*

*litated state,* which, it will just now appear is that in which the changes induced by position are the most remarkable." In four of these cases, the existence of hypertrophy with dilatation has been ascertained by post-mortem examination ; and of the other two, there can be no doubt of the state of the heart in one of them—while in the other, hypertrophy is more than probable. The following are the results of a great number of observations, made both in hospital and in private practice, upon the effect of posture on the pulse in various diseases.

" 1st. That the greatest difference occurs in patients labouring under fever, or in a debilitated state in consequence of fever or any other cause. It may amount to 30, 40, or even 50, between the horizontal and erect postures.

2dly. That this difference decreases after the first quarter of an hour in most cases, but always remains considerable, as long as the same position is observed.

3dly. That in persons not much debilitated the difference is much less than that stated above, and often does not amount to more than 10.

4thly. That when the patient lies down, the pulse rapidly falls to its former standard.

5thly. That in some the increase in frequency is greater between the horizontal and sitting posture, than between the latter and the erect; while in others the contrary takes place, so that generally the frequency in the sitting posture may be taken as a *mean.*

6thly. In persons convalescent from fever or acute diseases, I find it is extremely useful to the physician to ascertain the comparative frequency of the pulse in the horizontal and in the erect position. The greater the difference, the greater is the debility of the patient, *and consequently the more guarded must his medical attendant be in allowing him to sit up for any length of time,* particularly if the pulse on his lying down does not resume its usual degree of frequency."

Our author cannot conceive any plausible reason for this effect of posture on the pulse. Dr. Thomson, in his work on inflammation, adverts to this subject, and seems to have made a great many experiments to that effect.

